# Probiotic effects of *Lacticaseibacillus rhamnosus* 1155 and *Limosilactobacillus fermentum* 2644 on hyperuricemic rats

**DOI:** 10.3389/fnut.2022.993951

**Published:** 2022-09-30

**Authors:** Yanjun Li, Jun Zhu, Guodong Lin, Kan Gao, Yunxia Yu, Su Chen, Lie Chen, Zuoguo Chen, Li Li

**Affiliations:** ^1^College of Biosystems Engineering and Food Science, Zhejiang University, Hangzhou, China; ^2^Department of Research and Development, Hangzhou Wahaha Group Co., Ltd., Hangzhou, China; ^3^Key Laboratory of Food and Biological Engineering of Zhejiang Province, Hangzhou, China

**Keywords:** hyperuricemia, lactic acid bacteria, urate-lowering effect, intestinal excretion, xanthine oxidase, ABCG2, gut microbiota

## Abstract

Hyperuricemia is the main cause of gout and involved in the occurrence of multiple diseases, such as hypertension, metabolic disorders and chronic kidney disease. Emerging evidence suggests that lactic acid bacteria (LAB) have shown the beneficial effects on the prevention or treatment of hyperuricemia. In this study, the urate-lowering effect of two LAB strains, *Lacticaseibacillus rhamnosus* 1155 (LR1155) and *Limosilactobacillus fermentum* 2644 (LF2644) on hyperuricemic rats were investigated. A hyperuricemic rat model was induced by the intragastric treatment of potassium oxonate, combined with a high purine diet. The oral administration of LR1155, LF2644, or a combination of LR1155 and LF2644 for 4 weeks significantly prevented the rise of the serum uric acid (UA) induced by hyperuricemia. LR1155 and LF2644 significantly elevated the fecal UA levels, increased the UA content and up-regulated gene expression of UA transporter, ATP-binding cassette subfamily G-2 (ABCG2), in colon and jejunum tissues, suggesting the accelerated UA excretion from the intestine. Besides, LR1155 significantly inhibited the activity of xanthine oxidase (XOD) in liver and serum, benefited the reduce of UA production. In addition, LF2644 strengthened the gut barrier functions through an up-regulation of the gene expressions for occluding and mucin2, accompanied with the reduced inflammatory indicators of lipopolysaccharide (LPS) and interleukin-1β (IL-1β) in hyperuricemic rat. Moreover, using 16s rDNA high-throughput sequencing of feces, LR1155 was shown to improve the hyperuricemia induced gut microbial dysbiosis. The genera *Roseburia*, *Butyricicoccus*, *Prevotella*, *Oscillibacter*, and *Bifidobacterium* may associate with the effect of LR1155 on microbiota in hyperuricemic rats. Collectively, the results indicated that LR1155 and LF2644 exhibit urate-lowering effects and could be used alone or in combination as a new adjuvant treatment for hyperuricemia.

## Introduction

Hyperuricemia is a metabolic disease caused by accumulated uric acid (UA) (1), that results in many diseases including gout, cardiovascular disease, metabolic syndrome, and chronic kidney disease (2). In recent decades, hyperuricemia has become a prevalent disease with increasing incidence and it is thought to be linked with the development of the economy and unhealthy lifestyle (3). In view of rapid economic developing countries, such as China, the prevalence rate of hyperuricemia has increased noticeably (4).

The homeostasis of UA level depends on the balance of its production and excretion rate. In the case of UA synthesis, xanthine oxidase (XOD) is the crucial enzyme catalyzing purine conversion to UA in humans (5). This enzyme is distributed mainly in the liver, intestine, kidney, as well as plasma and is involved in two stages of UA generation: conversion of hypoxanthine to xanthine and subsequently xanthine to UA (6). On the other side, the excretion of UA is mainly through two pathways, the renal pathway and the extra-renal pathway. It is reported that approximately 70% of the UA is excreted through the renal pathway, while the remaining 30% is excreted by feces from the intestine (7). Although most of the excretion occurs in the kidney, the intestinal secretion is also testified to be an important alternative pathway, especially in renal insufficient patients, for example, patients with diabetic nephropathy or cardiorenal disease (8, 9). Compared to the renal pathway, fewer studies were reported on intestinal excretion and less was studied on the corresponding transporters. Recent researches reported that ATP-binding cassette subfamily G-2 (ABCG2), also known as breast cancer resistance protein, is a high-volume UA transporter located in the intestinal epithelial cells (10, 11). The dysfunction of ABCG2 reduces the excretion of UA in the intestinal tract, resulting in increased plasma UA levels (12).

Correspondingly, current anti-hyperuricemia drugs mainly include XOD inhibitors, like allopurinol and febuxostat (13), and uricosuric drugs, like benzbromarone (14). Allopurinol is recommended as the first-line treatment for hyperuricemia. However, it is reported to have many side effects, including allergies, gastrointestinal discomfort, myelosuppression and toxic epidermal necrolysis (13, 15). Febuxostat and benzbromarone have also been reported to be associated with serious hepatotoxicity (14, 16). Since the safety and tolerability problems of the current medications still exist, intensive research is ongoing to look for new strategies for the treatment of hyperuricemia.

Recent studies have revealed that the gut microbiota composition of gout patients is highly distinct from healthy individuals and the gut microbiota participate in the metabolism of purine and UA, which might provide a new angle for anti-hyperuricemic treatment (17, 18). Probiotic microorganisms are well-known for their beneficial effects in modulating the intestinal flora and improving gut barrier integrity (19, 20). Strains of lactic acid bacteria (LAB) have a long history of safe and effective use as probiotics (20). Studies suggested that several LAB strains, such as *L.brevis* DM9218 (21) or *L. gasseri* PA-3 (22), are capable of taking up and incorporating purine nucleoside to ameliorate hyperuricemia. Gut microbiota dysbiosis caused by hyperuricemia was regulated by supplementation of *L.brevis* DM9218 or *L. fermentum* JL-3 (23, 24). Moreover, microbial fermented extracts of *Lactobacillus* were reported to prevent the UA elevation by inhibiting the XOD activity (25). However, whether the LAB strains may play a role in the aspect of UA excretion effects has not been clarified. Previously, we isolated two LAB strains, *Lacticaseibacillus rhamnosus* 1155 (LR1155) and *Limosilactobacillus fermentum* 2644 (LF2644), from traditional fermented dairy products which showed great capacities for assimilating purine nucleosides (guanosine) and exhibited good potential in hyperuricemia prevention. In the present study, it was aimed to evaluate the anti-hyperuricemic effects of LR1155 and LF2644, singly or in combination, and then to investigate their underlying mechanisms.

## Materials and methods

### Preparation of lactic acid bacteria strains

LR1155 and LF2644 were isolated from traditional fermented dairy products originating from districts in Xinjiang and Inner Mongolia, China. Both strains were preserved at the China General Microbiological Culture Collection Center (CGMCC, Beijing, China) with the preservation numbers of CGMCC 11955 (LR1155) and CGMCC 16754 (LF2644).

For functional assessment, each strain was grown in de Man Rogosa-Sharpe (MRS) broth (Oxoid, Hants, UK) and cultured anaerobically at 37^°^C until the end of the log phase. Strains were collected by centrifugation (4,000 × g, 10 min, 4^°^C), freeze-dried and smashed to the powder, and the number of viable cells was determined using a plate assay. The powder of LR1155 and LF2644 was stored at –20^°^C and re-suspended in 0.9% saline solution before the animal test. To prepare the heat-killed bacteria, the suspension of each strain was autoclaved at 95^°^C for 10 min and was then plated on MRS agar to confirm that no viable cells remained.

### Animals and experimental design

The design and procedures involving the rats in this study were carried out in full accordance with the requirement of the “Governing Regulation for the Use of Experimental Animals” in Zhejiang Province, China. All the animal experimental procedures were approved by the Animal Care and Use Committee of Hangzhou Wahaha Tech Co., Ltd. (Ethics Code Permit WHH2020002). Ninety-six male *SD* rats, weighing 200–220 g (Shanghai SLAC Laboratory Animal Co., Ltd., Shanghai, China) were maintained under a 12-h day-night cycle at room temperature of 22–24^°^C, with free access to food and water.

**FIGURE 1:**
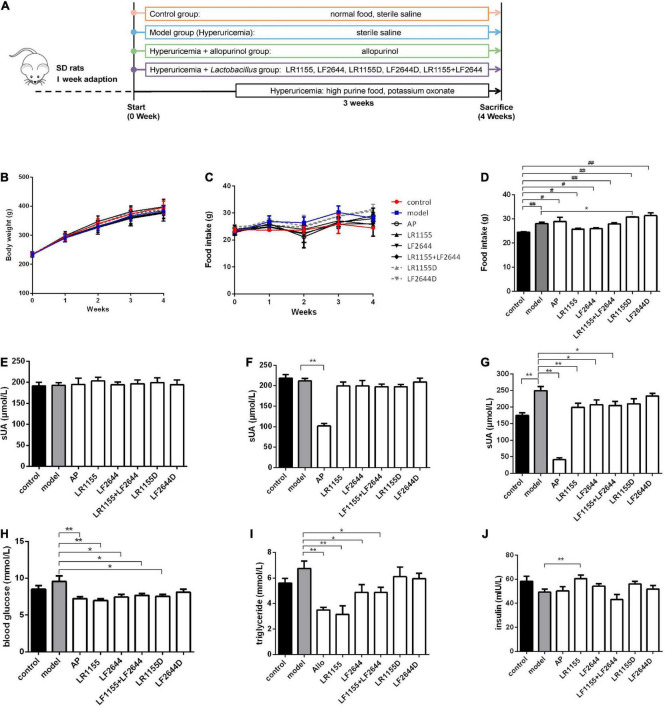
Effects of LR1155 and LF2644 on body weight, food intake and metabolic parameters in hyperuricemia rats. **(A)** The timeline of the rat experiment. **(B)** Body weight and **(C)** food intake during the experiment. **(D)** Food intake on week 4. **(E–G)** Serum UA levels on weeks 0, 1, and 4. **(H)** Blood glucose, **(I)** triglyceride and **(J)** insulin levels at the end of the experiment. PO, potassium oxonate; AP, allopurinol; LR1155D and LF2644D, heat-killed LR1155 and LF2644. Data are expressed as mean ± SEM (*n* = 10–12 per group). *P < 0.05, **P < 0.01 vs. the model group, ^#^*P* < 0.05, ^##^*P* < 0.01 vs. the control group.

After 1 week of acclimatization to the housing facility, the rats were randomly divided into 8 groups: control, model (hyperuricemia), AP (allopurinol, 20 mg/kg BW/d), LR1155 (live LR1155, 1.5 × 10^8^ CFU/d), LF2644 (live LF2644, 1.5 × 10^8^ CFU/d), LR1155D (heat-killed LR1155, 1.5 × 10^8^ CFU/d), LF2644D (heat-killed LF2644, 1.5 × 10^8^ CFU/d), and LR1155 + LF2644 (a combination of live LR1155 and LF2644, 7.5 × 10^7^ CFU/d each). The timeline of the rat experiments in this study is shown in Figure 1A. To establish the hyperuricemia models, rats in all groups, except the control group, were given high purine food and intragastrically administrated potassium oxonate at 300 mg/kg once per day for a total of 3 weeks. The high purine diet (per 100 g) contains 20 g yeast extract and 0.1 g adenine. Allopurinol or bacteria were suspended in 0.9% saline solution, administrated intragastrically 1 week before the hyperuricemia was induced, and continued for 3 weeks. The control group and model group were treated with an equal saline solution *via* the intragastric route.

### Sample collection

Body weight and food intake were recorded weekly. Blood samples were taken from the tail vein of rats after a 12 h fasting at week 0 and week 1. At the end of the experiment, rats were sacrificed by neck dislocation under ether anesthesia, and whole blood samples were collected from cardiac punctures. The serum was separated by centrifugation at 3,000 g for 10 min. Liver, jejunum, ileum and colon samples were collected, rinsed with phosphate buffered saline (PBS), and immediately frozen at –80^°^C. Part of the kidney tissues was fixed for hematoxylin and eosin (H&E) staining. Urine samples were collected at 0, 1, and 4 w (before the rats were sacrificed). Fresh feces were collected before the end of the experiment. All the samples were stored at –80^°^C until use.

### Biochemical analysis

The urine samples were centrifuged at 3,000 g for 10 min and the supernatants were collected. The liver and colon tissues were mixed with normal saline with a ratio of 1:9 on ice, shocked by the TissuePrep (Jieling Instrument Manufacturing Co., Tianjin, China), and then centrifuged at 8,000 g for 10 min to obtain the tissue homogenate. The bicinchoninic acid total protein assay kit (Beyotime Biotechnology, Shanghai, China) was used to quantify the protein concentration in the tissue homogenate. The levels of glucose, triglyceride, insulin, creatinine, urea nitrogen, interleukin-1β (IL-1β), interleukin-10 (IL-10), and lipopolysaccharide (LPS) in serum, UA in serum and urine, and enzymatic activities of XOD in serum and tissues were measured using commercial kits (Nanjing Jiancheng Biotechnology Institute, Nanjing, China) according to the manufacturer’s instructions.

### Histopathological examination

The renal tissues were fixed in 5% paraformaldehyde solution, dehydrated with ethanol and embedded in paraffin. Samples were prepared in 4 μm thick paraffin sections and stained by the H&E method. The pathological changes in renal tissue were observed under a microscope (Olympus, Tokyo, Japan).

### Determination of uric acid in feces and tissues

UA analysis in feces was performed by high-performance liquid chromatography (HPLC) (8, 26, 27). 50 mg freeze-dried, pulverized rat stool was mixed with PBS solvent, and homogenized ultrasonically. Then the extracted supernatant was obtained by centrifugation at 4,000 g, 4^°^C for 10 min, and filtered using a 0.45 μm filter. The HPLC system was made up of an Agilent 1,200 Series with an ultraviolet detector (Agilent Technologies, Waldbronn, Germany). Samples were separated under the following conditions: column, Reversed-phase C18 (4.6 mm × 250 mm, 5 μm); mobile phase, 0.2% acetic acid mixed with methanol (94:6); flow rate, 1 mL/min; column temperature, 30^°^C; and detector wavelength, 288 nm.

The tissues of jejunum, ileum and colon were homogenized in ice-cold PBS solvent (pH 7.4), shocked by the TissuePrep to obtain a 10% (w/v) homogenate, and were subsequently centrifuged at 12,000 g for 10 min (28, 29). The supernatants were collected and the levels of UA were detected by a commercial kit provided by Nanjing Jiancheng Biotechnology Institute (China). The protein concentration in the supernatant was analyzed and the result was expressed as UA production (μmol) per mg tissue protein.

### RNA isolation and real-time PCR analysis

Briefly, total RNA was extracted from the colon and jejunum tissues using Trizol reagent following the manufacturer’s protocol (Invitrogen, CA, USA). The synthesis of first-strand cDNA was carried out using the PrimeScript transcription reagent kit (Takara, Shiga, Japan). Quantitative real-time PCR was performed using the SYBR^®^ Premix Ex Taq II kit (Takara, Shiga, Japan) according to the manufacturer’s protocols. The primer sequences are provided in Table 1. The experimental data were normalized to a housekeeping gene, glyceraldehyde-3-phosphate dehydrogenase (GAPDH). Cycle threshold (Ct) values were monitored, and the relative gene expressions were analyzed using the 2^–ΔΔCt^ method (30).

**TABLE 1:** Primers used in this study.

Products	Forward primer (5’→3’)	Reverse primer (5’→3’)	bp
Occludin	CTACTCCTCCAACGGCAAAG	AGTCATCCACGGACAAGGTC	118
MUC-2	GGCCACTGAGAACAGGATTG	CAGGCTCCTGAAGTGAATGTC	184
ZO-1	GAGGATGTGCACGATCCAAG	CAGGACAACATCCCCTTC	291
GLUT9	CAAAGAACTGGTCCTGCTCG	CGTCCCACAATGAGCATCTC	367
ABCG2	GGCCTGGACAAAGTAGCAGA	GTTGTGGGCTCATCCAGGAA	137
GAPDH	AATGCATCCTGCACCACCAA	GTAGCCATATTCATTGTCATA	516

### 16s rDNA sequencing and data analysis

Total DNA from each sample of fecal materials was extracted using the E.Z.N.A Stool DNA kit (Omega Bio-Tek, GA, USA) according to the manufacturer’s protocol. To balance the necessary amount of replicates and the cost, six samples per group were selected randomly for 16s rDNA high-throughput sequencing. The V3-V4 region of the bacterial 16S rDNA gene were amplified using a specific forward primer (5’-CCTACGGGNGGCWGCAG-3’) and a reverse primer (5’-GACTACHVGGGTATCTAATCC-3’). Amplicons were purified using AMPure XT beads (Beckman Coulter Genomics, Danvers, MA, USA) and quantified by Qubit (Invitrogen, CA, USA) according to manufacturer’s instructions. Illumina NovaSeq platform was used for sequencing the purified amplicons. The raw reads were deposited into the NCBI Sequence Read Archive (SRA) database (PRJNA838820).

Quality filtering on the raw reads was performed under specific filtering conditions to obtain the high-quality clean tags according to the Fqtrim (v0.94). Chimeric sequences were filtered using Vsearch software (v2.3.4). Feature table and feature sequence were obtained after dereplication using DADA2. The blast was used for sequence alignment, and the feature sequences were annotated with the SILVA database for each representative sequence. The results were visualized using R software (v4.0.3) and GraphPad Prism (v9.0).

### Statistical analysis

Statistical analyses were performed using SPSS 20.0. *P*-value less than 0.05 was considered significant. Data were analyzed using one way analysis of variance (ANOVA) followed by Turkey’s *post-hoc* test. The significant differences between two groups were assessed by independent sample *t*-test. Microbial data analysis was performed using QIIME 2 and R software (v4.0.3). The *q* value was calculated based on the *P*-value with false discovery rate (FDR) correction. A *q*-value < 0.05 was regarded as statistically different.

## Results

### Body weight, food intake and metabolic parameters in hyperuricemia rats

Animal experiments were conducted as shown in Figure 1A. Following the treatment with either LAB strains or allopurinol by gavage once daily for 4 weeks, there was no significant change in the body weight among different groups (Figure 1B, *P* > 0.05). Compared with the control group, the daily food intake was significantly increased in the hyperuricemia model group on week 4 (Figures 1C,D, *P* < 0.05), while no significant difference was observed among the hyperuricemia-induced groups (*P* > 0.05) except for the LR1155D group.

Blood samples were taken on weeks 0, 1, and 4, and the serum UA was detected. At baseline, all groups had comparable values of serum UA (Figure 1E, *P* > 0.05). Before the hyperuricemia was induced, oral treatment with allopurinol significantly decreased the serum UA (Figure 1F, *P* < 0.01), but no changes were observed in LAB treated groups (*P* > 0.05). At the end of the experiment, after 3 weeks of a high purine diet combined with administrating of potassium oxonate, the serum UA level of hyperuricemia rats increased to 249.78 ± 42.85 μmol/L, which was significantly elevated compared to the control rats (Figure 1G, *P* < 0.01). The administration of live LR1155, LF2644 or a combination of live LR1155 and LF2644 significantly prevented the rise of serum UA by 17.0–20.2%, although not as much as allopurinol did. Rats treated with heat-killed LAB strains observed no significant difference in serum UA compared with the model group (*P* > 0.05), indicating only living bacteria have urate-lowering capabilities. Emerging evidence suggests that serum uric acid is associated with metabolic diseases and its components such as hyperglycemia and hypertrilyceridaemia (31). Interestingly, an evident decrease in serum glucose and triglyceride was detected in allopurinol (Figures 1H,I, *P* < 0.01), LR1155 (*P* < 0.01), LF2644 (*P* < 0.05), and two strains combined groups (*P* < 0.05) when compared to the model group. An elevated serum insulin level of the LR1155 group (Figure 1J, *P* < 0.01) compared with the model group may partly explain the decrease in serum glucose level.

### Effects of LR1155 and LF2644 on xanthine oxidase activities

**FIGURE 2:**
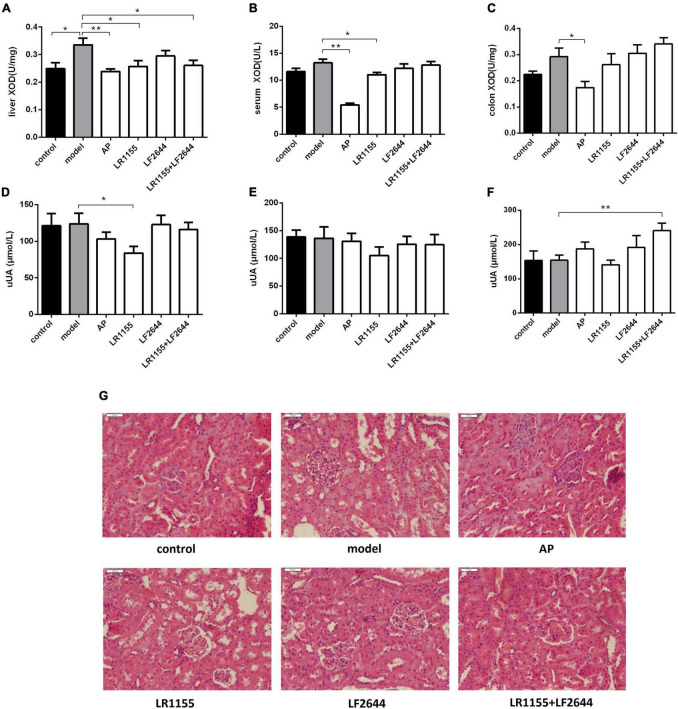
Effects of LR1155 and LF2644 on XOD activities, urine UA levels and renal histology in hyperuricemia rats. **(A–C)** Activities of XOD in liver, serum and colon. **(D–F)** Urine UA levels on weeks 0, 1, and 4. **(G)** Kidney tissues by H&E stain observed at a magnification of 200 ×. Data are expressed as mean ± SEM (*n* = 10–12 per group). *P < 0.05, **P < 0.01 vs. the model group.

Blocking the UA synthesis by reducing the activity of XOD is a key strategy for the treatment of hyperuricemia. As shown in Figure 2A, the enzymatic activity of XOD in the liver was significantly increased in the model group as compared to the control (*P* < 0.05). Allopurinol, commonly known as an XOD inhibitor, significantly suppressed the XOD activity levels in liver (Figure 2A, *P* < 0.01), serum (Figure 2B, *P* < 0.01) and colon tissues (Figure 2C, *P* < 0.05) when compared with the hyperuricemia model. Administration of LR1155 also significantly decreased the XOD activities in the liver and serum (*P* < 0.05). The results indicated that treatment with LR1155 opposed the UA increase *via* the reduction of XOD activity.

### Effects of LR1155 and LF2644 on urine uric acid and renal functions

To confirm whether the anti-hyperuricemia activities of LAB strains were attributed to their uricosuric effects, we detected the urine UA on weeks 0, 1, and 4, and the results were summarized in Figures 2D–F. At the end of the experiment, the separated LR1155 or LF2644 showed no obvious effect compared with the model group, but the urine UA was significantly increased when the rats were treated with a combination (Figure 2F, *P* < 0.01). Impaired renal excretion of UA was considered to be one of the causes of hyperuricemia (32). In this hyperuricemia model, the urine UA showed no significant change when compared with the control group (Figure 2F, *P* > 0.05). The histological results (Figure 2G) revealed that a high-purine diet and potassium oxonate did not cause obvious damage in renal ultra-structures, only the glomeruli showed a slight capsular synechia, and this might be due to the limited modeling time. Administration of two LAB strains had no significant influence on the morphologic structures of glomeruli and renal tubules.

### Effects of LR1155 and LF2644 on intestinal uric acid excretion

**FIGURE 3:**
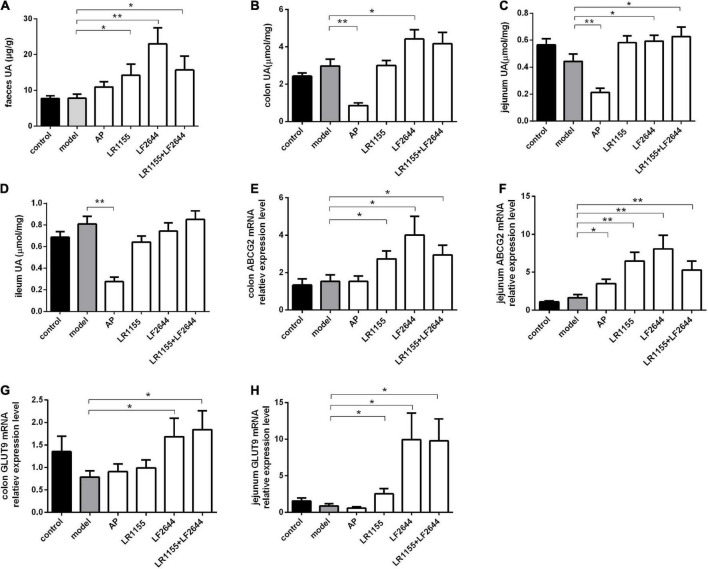
Effects of LR1155 and LF2644 on intestinal uric acid excretion in hyperuricemia rats. **(A)** Fecal UA levels. **(B–D)** UA contents of colon, jejunum and ileum. **(E,F)** Gene expressions of ABCG2 in colon and jejunum. **(G,H)** Gene expressions of GLUT9 in colon and jejunum. Data are expressed as mean ± SEM (*n* = 10–12 per group). *P < 0.05, **P < 0.01 vs. the model group.

Furthermore, we measured the UA contents in feces and different intestinal segments, including the colon, jejunum and ileum, to evaluate the intestinal UA excretion in hyperuricemia rats. As shown in Figure 3A, no significant differences (*P* > 0.05) were observed in the fecal UA levels between the model groups and control group. However, the fecal UA levels of rats in three LAB-treated groups (LR1155, LF2644, and LR1155 + LF2644) were remarkably increased (*P* < 0.05) in comparison to hyperuricemic rats. Similarly, when compared with control rats, tissue UA content in hyperuricemia rats did not show significant changes (Figures 3B–D, *P* > 0.05). However, UA levels in colon and jejunum tissues were higher (*P* < 0.05) in LF2644-treated rats than those in hyperuricemia model rats. Combined administration of LR1155 and LF2644 also elevated the UA content in jejunum tissues (*P* < 0.05). As a positive control, allopurinol showed a remarkable reduction in serum UA levels but there was no significant change (Figure 3A, *P* > 0.05) in fecal UA levels compared with the hyperuricemic rats, and the UA levels in different intestinal segments were even lower (Figures 3B–D, *P* < 0.01) than those in the model groups.

Effects of LAB strains on ABCG2 mRNA expressions of colon and jejunum are shown in Figures 3E,F, from which it can be seen that administration of LR1155, LF2644 or a combination of LR1155 and LF2644 significantly up-regulated the ABCG2 mRNA expressions in the colon (Figure 3E, *P* < 0.05) and jejunum (Figure 3F, *P* < 0.01) tissues when compared with the model group. Interestingly, the f old change of the relative gene expression level of ABCG2 was found to be higher in the jejunum than that in the colon tissues by administration of LR1155 (4.0 vs. 1.8), LF2644 (5.0 vs. 2.6) or a combined LR1155 and LF2644 (3.2 vs. 1.9). In addition, as shown in Figures 3G,H, LF2644 (*P* < 0.05) or combined LR1155 and LF2644 (*P* < 0.05) markedly up-regulated glucose transporter 9 (GLUT9) gene expressions in colon and jejunum tissues as compared with the hyperuricemia rats.

### Effects of LR1155 and LF2644 on inflammation and intestinal barrier function

**FIGURE 4:**
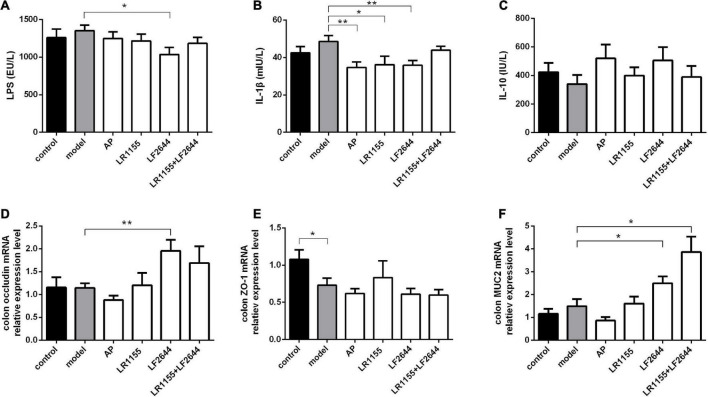
Effects of LR1155 and LF2644 on inflammatory cytokines and intestinal barrier function in hyperuricemia rats. **(A–C)** Serum LPS, IL-1β and IL-10 levels at the end of the experiment. **(D–F)** Gene expressions of occluding, ZO-1 and MUC2 in colon tissue. Data are expressed as mean ± SEM (*n* = 10–12 per group). *P < 0.05, **P < 0.01 vs. the model group.

The findings on the effects of inflammation and intestinal barrier function of hyperuricemia rats challenged with two LAB strains were obtained and shown in Figure 4. LF2644 showed better performance in comparison to LR1155. Administration of LF2644 significantly reduced the LPS (Figure 4A, *P* < 0.05) and IL-1β (Figure 4B, *P* < 0.01) levels when compared with the model group, despite no obvious changes being detected between the model and control group. Moreover, supplementation with LF2644 was found to improve the gut barrier function as the gene expressions of occludin (Figure 4D, *P* < 0.01) and mucin 2 (Figure 4F, *P* < 0.05) were higher in the LF2644 group than those in the model and control group.

### Gut microbiota composition of hyperuricemia rat was modulated by LR1155

**FIGURE 5:**
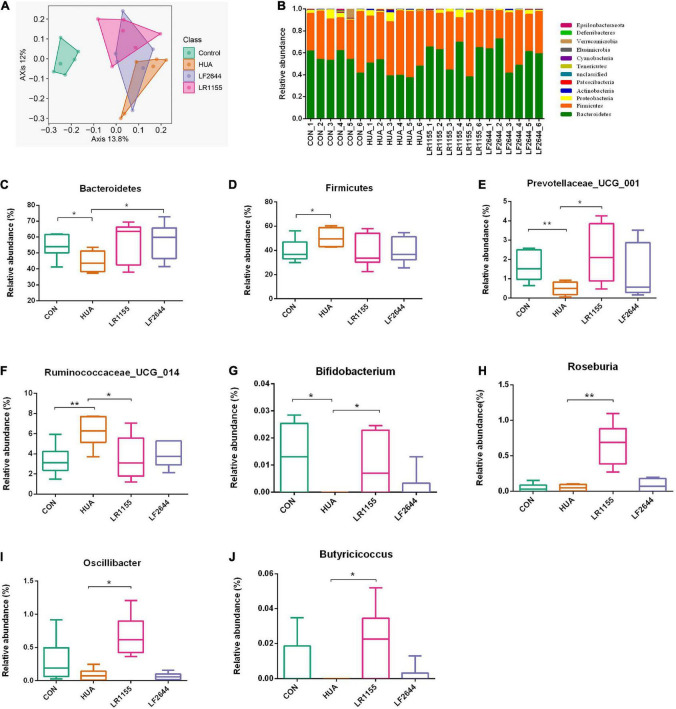
Effects of LR1155 and LF2644 on the intestinal microbial composition of hyperuricemia rat. **(A)** Principle-coordinates analysis (PCoA) based on the Bray-Curtis distances among different samples. **(B)** Microbial community structure indicated by the percentile difference of bacteria at the phylum level. **(C,D)** The changes of gut microbial taxa at the phylum level (*Bacteroidetes* and *Firmicutes*). **(E–J)** The relative abundance of key differential genera. CON, control; HUA, hyperuricemia (model); *n* = 6; data are expressed as median with range (min to max). *P < 0.05, **P < 0.01 vs. the model group.

To assess the influences of LR1155 and LF2644 on intestinal flora in hyperuricemia rats, the 16s rDNA sequencing analysis was performed on the microbiota in feces. The Shannon diversity index didn’t show a significant difference among groups (data not show). The gut microbial beta diversity among the groups was evaluated by the Bray-Curtis distance-based principle-coordinates analysis (PCoA). The results implied a significant difference of gut microbiome composition between hyperuricemia and control groups. LR1155 supplementation showed distinct separations from both the control and hyperuricemia groups, thus diminished the effect of hyperuricemia on microbiota changes (Figure 5A). At the phylum level, the relative abundance in each sample of four detected groups was exhibited in Figure 5B. The dominant phyla of the gut microbiota in rats were *Bacteroidetes*, *Firmicutes*, *Proteobacteria*, and *Actinobacteria* (relative abundance > 0.5%). A significant decrease in the relative abundance of *Bacteroidetes* and an increase in *Firmicutes* were detected in the hyperuricemia group when compared to the control (Figures 5C,D, *P* < 0.05). Administration of LR1155 caused no significant changes in the abundances of *Bacteroidetes* and *Firmicutes* in comparison to the hyperuricemia group (*P* > 0.05). At the genus level, results showed that the relative abundance of *Prevotellaceae_UCG_001* (Figure 5E, *P* < 0.01) and *Bifidobacterium* (Figure 5G, *P* < 0.05) decreased after hyperuricemic modeling, while that of *Ruminococcaceae_UCG_014* increased (Figure 5F, *P* < 0.01). The alterations in the abundance of these genera were reversed to control levels following administration of LR1155 (*P* < 0.05). Moreover, the abundances of *Roseburia* (Figure 5H, *P* < 0.01), *Oscillibacter* (Figure 5I, *P* < 0.05) and *Butyricicoccus* (Figure 5J, *P* < 0.05) were significantly higher by LR1155 treatment than those in the hyperuricemia group. The results suggest that LR1155 may have several modulatory effects on the gut microbiota composition of hyperuricemia-induced rats.

## Discussion

Hyperuricemia is a metabolic disorder associated with gout, cardiovascular disease, renal injury and other comorbidities. Although this disease afflicts humans for years, the clinical drugs currently use for urate-lowering therapies are limited and most of them are considered to be associated with various adverse effects (13, 15). Probiotic treatment might be a promising strategy for anti-hyperuricemia intervention (22–25). In this study, we revealed that LR1155 and LF2644 isolated from Chinese traditional fermented dairy food exhibited an anti-hyperuricemia effect, and the underlying mechanism for each strain was testified.

The model of the hyperuricemic rat was established by a combination of a high purine diet and potassium oxonate, since rats usually express uricase, a hepatic enzyme which degrade UA to allantoin (33). As expected, rats fed with a high purine diet and potassium oxonate for 3 weeks induced hyperuricemia. Notably, the interventions of two LAB strains, LR1155 and LF2644, were able to significantly reduce the UA concentration at a dose of 1.5 × 10^8^ CFU/d, either alone or in combination.

The levels of serum UA are maintained by the balance between UA synthesis and excretion, so either decreased excretion or increased synthesis will lead to elevated UA concentrations (34). XOD plays an important role in converting xanthine and hypoxanthine into UA, therefore abnormal activity of XOD usually leads to excessive production of UA (5, 6). Intervention with LR1155 restored the increased XOD activity in the liver to normal level, which suggested that LR1155, but not LF2644, can inhibit the XOD activity to reduce the production of UA. Similar XOD-inhibiting effects were reported in microbial fermented extracts from *Lactobacillus* and *Acetobacter* (25), indicating the bacteria metabolites might be effective. The kidney has been recognized as a main regulatory site of serum UA in humans (12). Drugs like benzbromarone and probenecid are designed to enhance urate excretion *via* the renal pathway (13). However, current studies show that the intestine is also an important potential organ for the excretion of UA (11). In this study, the fecal UA levels were remarkably increased either in the use of LR1155 or LF2644 alone, or in combination. An elevated UA level in colon and jejunum tissues was also observed, suggesting the increased fecal UA levels might be related to the excessive exist of UA in these particular segments of the intestine. The results suggested that the intestinal excretion might be an alternative pathway for LR1155 and LF2644 to promote the excretion of UA.

UA-related transporters play an important role in the excretion of UA *via* intestinal epithelial cells. ABCG2 is a high-capacity UA transporter located on the apical membrane of enterocytes, the dysfunction of which raises hyperuricemia risk (12). Previous studies showed that both ABCG2 knockout mice and ABCG2 inhibiter, elacridar, caused decreased intestinal urate secretion and an increase in serum UA level (9, 12). Several prebiotics, such as chicory and stevia residue, are reported to promote the intestinal excretion of UA by enhancing the expression of ABCG2 (28, 35). GLUT9 is a reabsorption transporter of UA expressed in the kidney, which is responsible for regulating the UA back to the blood (2). However, the role of GLUT9 in intestinal handling of urate is ambiguous. The enterocyte-specific GLUT9 deficiency in mice impairs enterocyte urate transport and resulted in an increased UA in blood (8). It seems that GLUT9, located both in the basolateral and apical membranes in renal and intestinal epithelial cells, might be involved in urate reabsorption in the kidney but in urate excretion in the intestine (36). Our results demonstrated that LR1155 and LF2644 can promote the intestinal excretion of UA by upregulating the gene expressions of ABCG2 and GLUT9.

Previous studies have reported that the intestinal microbiota of gout patients was highly distinct from healthy individuals, suggesting the altered gut microbiota may associate with gout and may serve as a new target for disease treatment (17, 18). In the present study, hyperuricemia significantly changed the composition of the gut microbiota, with the decreased relative abundance of *Bacteroidetes* and increased that of *Firmicutes*, which is similar to previous research based on purine-induced hyperuricemia rats (37). LR1155 treatment improved the gut microbiota disturbed by hyperuricemia, but LF2644 had no obvious effect. At the genus level, *Roseburia* and *Butyricicoccus* are part of commensal bacteria producing short-chain fatty acids, especially butyrate, which affects motility, immunity maintenance and anti-inflammatory properties in the colon (38, 39). LR1155 has the potential to increase the *Roseburia* and *Butyricicoccus* abundance in rats, the same effect was detected in *Prevotella*, a propionate producer extensively present in the healthy human body (40). Moreover, we observed a drastic increase of *Oscillibacter* in LR1155-fed rats. A decline of *Oscillibacter* abundance was also reported when analyzing the association of individual microbial genera with obesity (41). Previous studies compared the composition of the gut microbiome in gout patients to healthy controls, and decreased fold change of *Oscillibacter*, *Butyricicoccus* and *Bifidobacterium* were detected (42). Among them, the genus *Bifidobacterium* was found to be reduced both in gout patients (42) and hyperuricemia rat models (43). In this study, *Bifidobacterium* was found to be depleted in hyperuricemia rats and LR1155 supplements elevated the decrease of *Bifidobacterium.* Thus, LR1155 could effectively improve the disturbed intestinal microbial community of hyperuricemic rats.

Gout, a developed form of hyperuricemia, is usually characterized by persistent low-grade inflammation (44), since the crystalline urate can activate the NLRP3 inflammasome and contribute to IL-1β activation (45). We detected the serum IL-1β levels in the high-purine diet and potassium oxonate-induced hyperuricemia rats. No significant difference was detected between the normal and hyperuricemia rats, which means the modeling process did not elicit significant inflammatory changes. However, administration of LF2644 was demonstrated to significantly reduce the IL-1β levels, which might benefit the subsequent courses. LPS, the major outer membrane compound of Gram-negative bacteria, usually known as a classic factor triggering inflammation (46) was also decreased by LF2644 supplementation. The damage to the gut barrier allows increased permeation of LPS into the systemic circulation (47). Probiotics are reported to strengthen gut barrier functions to avoid such permeation (19, 48). In this study, the protective effect of LF2644 on gut barrier functions was observed. Occludin, an important transmembrane protein that constitutes the tight junction complex and links adjacent epithelial cells (49), was increased in colon tissues by treatment of LF2644. Mucin2, the main component of the intestinal mucus and also the well characterized secreted mucin of the gastrointestinal tract (50), significantly increased in the LF2644 group, providing additional evidence to support the reinforced barrier function by LF2644.

In summary, both LR1155 and LF2644 exhibited an anti-hyperuricemia effect, but each strain showed different properties. LR1155 can inhibit the XOD activity to reduce the production of UA and regulate the dysbiosis of gut microbiota caused by hyperuricemia. LF2644 strengthened the gut barrier functions, thus reducing the potential of LPS-triggered inflammation. Both strains promoted the intestinal excretion of UA by upregulating the UA transporters in enterocytes. Therefore, LR1155 and LF2644 can be used alone or in combination for the prevention of hyperuricemia. Current drugs for promoting UA excretion mainly concentrated their target on the kidneys, which might increase the burden on the kidneys, especially for patients with chronic kidney disease (11). LR1155 and LF2644 exerted their effects in accelerating the intestinal excretion of UA providing a promising alternative way of lowering UA. The results indicated that probiotics can be a promising strategy for preventing or alleviating hyperuricemia.

## Data availability statement

The datasets presented in this study can be found in online repositories. The names of the repository/repositories and accession number(s) can be found below: the NCBI Sequence Read Archive (SRA) database – PRJNA838820.

## Ethics statement

This animal study was reviewed and approved by the Animal Care and Use Committee of Hangzhou Wahaha Group Co., Ltd.

## Author contributions

JZ and YL designed the research and wrote the manuscript. JZ, GL, YY, LC, and ZC performed the experiments. JZ, GL, and LL collected the sample and data. JZ and KG analyzed the data. KG revised the manuscript. YL and SC provided the resources. All authors read and approved the final manuscript.
